# Validation and feasibility of liver T1 mapping using free breathing MOLLI sequence in children and young adults

**DOI:** 10.1038/s41598-020-74717-2

**Published:** 2020-10-27

**Authors:** Yeon Jin Cho, Woo Sun Kim, Young Hun Choi, Seul Bi Lee, SeungHyun Lee, Jung-Eun Cheon, MunYoung Paek, SeungTae Woo

**Affiliations:** 1 Department of Radiology, Seoul National University Hospital, 101 Daehak-ro, Jongno-gu, Seoul, 03080 Republic of Korea; 2 Department of Radiology, Seoul National University College of Medicine, 103 Daehak-ro, Jongno-gu, Seoul, 03080 Republic of Korea; 3 Institute of Radiation Medicine, Seoul National University Medical Research Center, 103 Daehak-ro, Jongno-gu, Seoul, 03080 Republic of Korea; 4 Siemens Healthcare Ltd., 23, Chungjeong-ro, Seodaemun-gu, Seoul, 03737 Republic of Korea; 5 Radiology, Bayer Korea Ltd., 23, Boramae-ro, Dongjak-gu, Seoul, 07071 Republic of Korea

**Keywords:** Liver, Paediatric research

## Abstract

We investigated the feasibility of free-breathing modified Look-Locker inversion recovery (MOLLI) sequence for measuring hepatic T1 values in children and young adults. To investigate the accuracy and the reproducibility of the T1 maps, a phantom study was performed with 12 different gadoterate meglumine concentrations and the T1 relaxation times of phantoms measured with the MOLLI sequence were compared against those measured with three different sequences: spin-echo inversion recovery, variable flip angle (VFA), and VFA with B1 correction. To evaluate the feasibility of free-breathing MOLLI sequence, hepatic T1 relaxation times obtained by free-breathing and breath-hold technique in twenty patients were compared. The phantom study revealed the excellent accuracy and reproducibility of MOLLI. In twenty patients, the mean value of hepatic T1 values obtained by free-breathing (606.7 ± 64.5 ms) and breath-hold (609.8 ± 64.0 ms) techniques showed no significant difference (p > 0.05). The Bland–Altman plot between the free-breathing and breath-hold revealed that the mean difference of T1 values was − 3.0 ms (− 0.5%). Therefore, T1 relaxation times obtained by MOLLI were comparable to the values obtained using the standard inversion recovery method. The hepatic T1 relaxation times measured by MOLLI technique with free-breathing were comparable to those obtained with breath-hold in children and young adults.

## Introduction

The increasing prevalence of chronic liver disease in children has a significant impact on public health^1^. Numerous conditions such as congenital disorders, infections, and inflammation can cause end-stage liver disease and hepatic fibrosis^2^. Liver parenchymal biopsy is the gold standard for the diagnosis and staging of hepatic fibrosis; however, needle biopsy is an invasive procedure that may result in various complications including hemorrhage and infection. Additionally, inter- or intra-observer variability and sampling errors may occur in needle biopsy^3^. Recently, various non-invasive and clinically-accepted methods for evaluating liver fibrosis have been developed. These include fibrosis scoring system using laboratory results, transient elastography, acoustic radiation force impulse imaging, magnetic resonance elastography, and T1 mapping using MRI^4^. Among these methods, T1 mapping is the method of quantifying the T1 relaxation time of the target tissue. T1 relaxation time depends on the composition of the tissue, and a prolonged T1 relaxation time usually reflects diffuse tissue fibrosis. It has already been established that hepatic T1 relaxometry is related to the extent of hepatic fibrosis or cirrhosis^5–10^. The methods of measuring tissue T1 relaxation time include (1) inversion recovery (IR) T1 mapping, which is the reference standard for T1 mapping, (2) variable flip angle (VFA) T1 mapping, (3) VFA with B1 inhomogeneity correction, and (4) look-locker and modified look-locker inversion recovery (MOLLI) sequence^6–8,11^. The most accurate method of measuring T1 relaxation time is through the acquisition of a series of inversion recovery pulse images with different inversion times. However, this technique is clinically impractical, especially in children, because it requires a relatively long scan time and multiple breath-holds^12^. MOLLI sequence was originally proposed for measuring T1 values of myocardium in cardiac imaging^13^. Since MOLLI can generate high resolution T1 maps in single breath-hold with good reproducibility, there have been several attempts to apply MOLLI sequence to obtain hepatic T1 maps in previous studies^8,14,15^. However, theses previous studies about hepatic T1 mapping using the MOLLI sequence have been conducted in adult patients with a breath-hold^5,8,16^. There have been a few recent studies in children that have attempted hepatic T1 mapping^17,18^. However, these studies were performed hepatic T1 mapping with breath-hold method. For universal usage of hepatic T1 mapping including children and adults patients with respiratory difficulty, evaluation of accuracy and feasibility of free breathing hepatic T1 mapping is required.

The purpose of our study is therefore to evaluate the usefulness of MOLLI sequence for measuring hepatic T1 values in comparison to the standard spin-echo inversion recovery (SE-IR) sequence and variable flip angle (VFA) sequence. We also aim to assess the feasibility of a free-breathing MOLLI sequence in children and young adults^8,19–21^.

## Results

### Phantom

**Figure 1:**
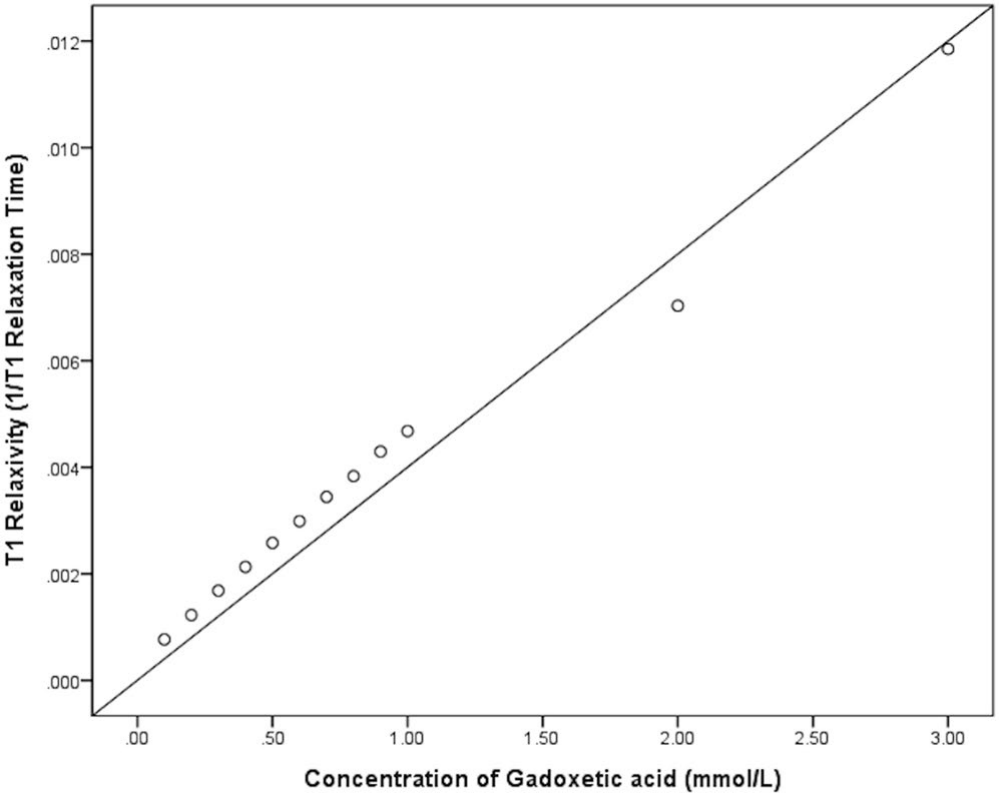
Correlation plot between 12 different concentrations of gadoterate meglumine and the T1 relaxation time. The Pearson correlation coefficient was 0.993 (p < 0.001).

**Figure 2:**
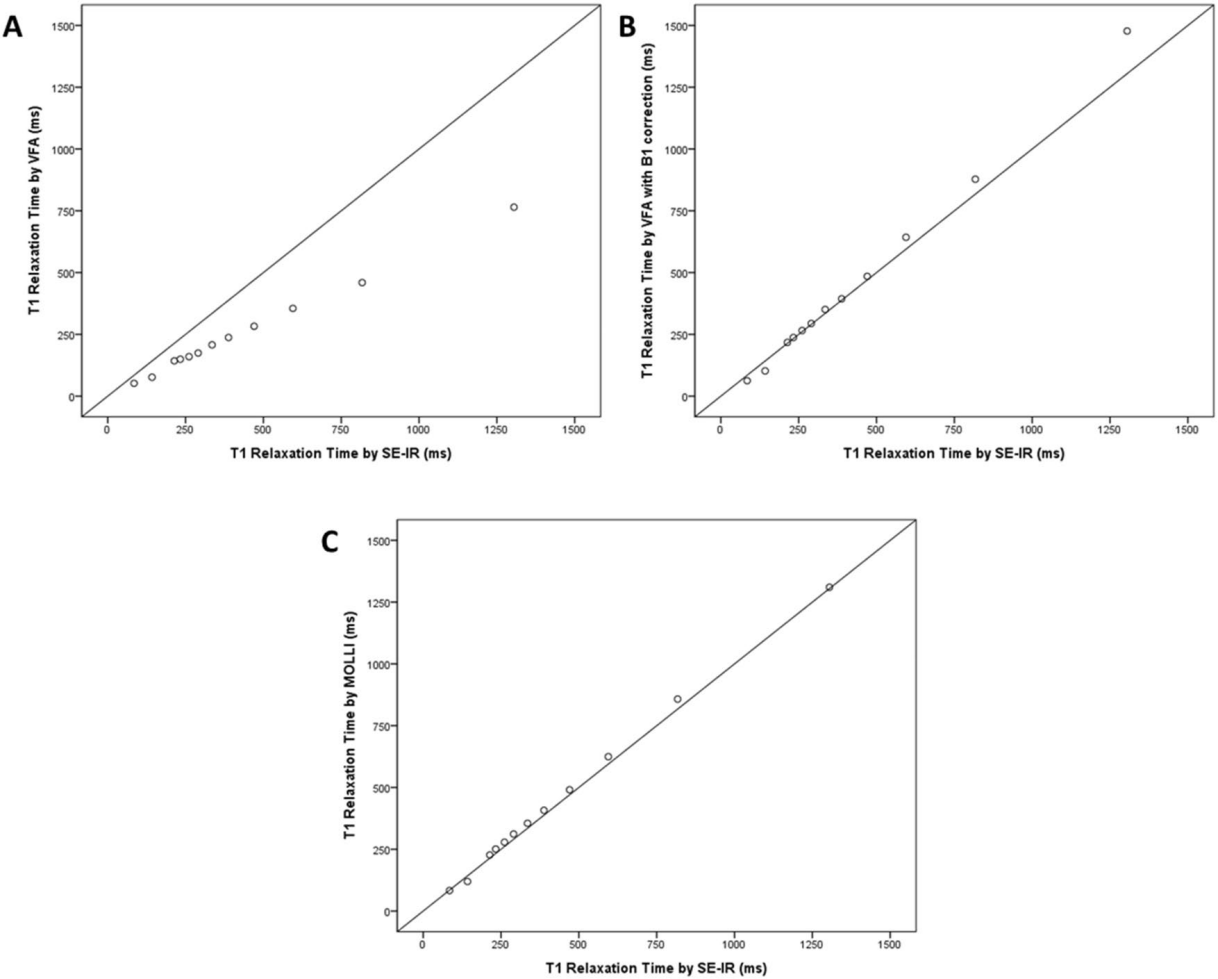
Correlation plot between T1 relaxation time measured in phantoms with VFA, VFA with B1 correction, and MOLLI versus standard SE-IR. (**A**) Correlation plot between T1 relaxation time measured with VFA and SE-IR (Pearson correlation coefficient = 0.999, ICC = 0.845). (**B**) Correlation plot between T1 relaxation time measured with VFA with B1 correction and SE-IR (Pearson correlation coefficient = 0.999, ICC = 0.994). (**C**) Correlation plot between T1 relaxation time measured with MOLLI and SE-IR (Pearson correlation coefficient = 0.999, ICC = 0.999).

**Figure 3:**
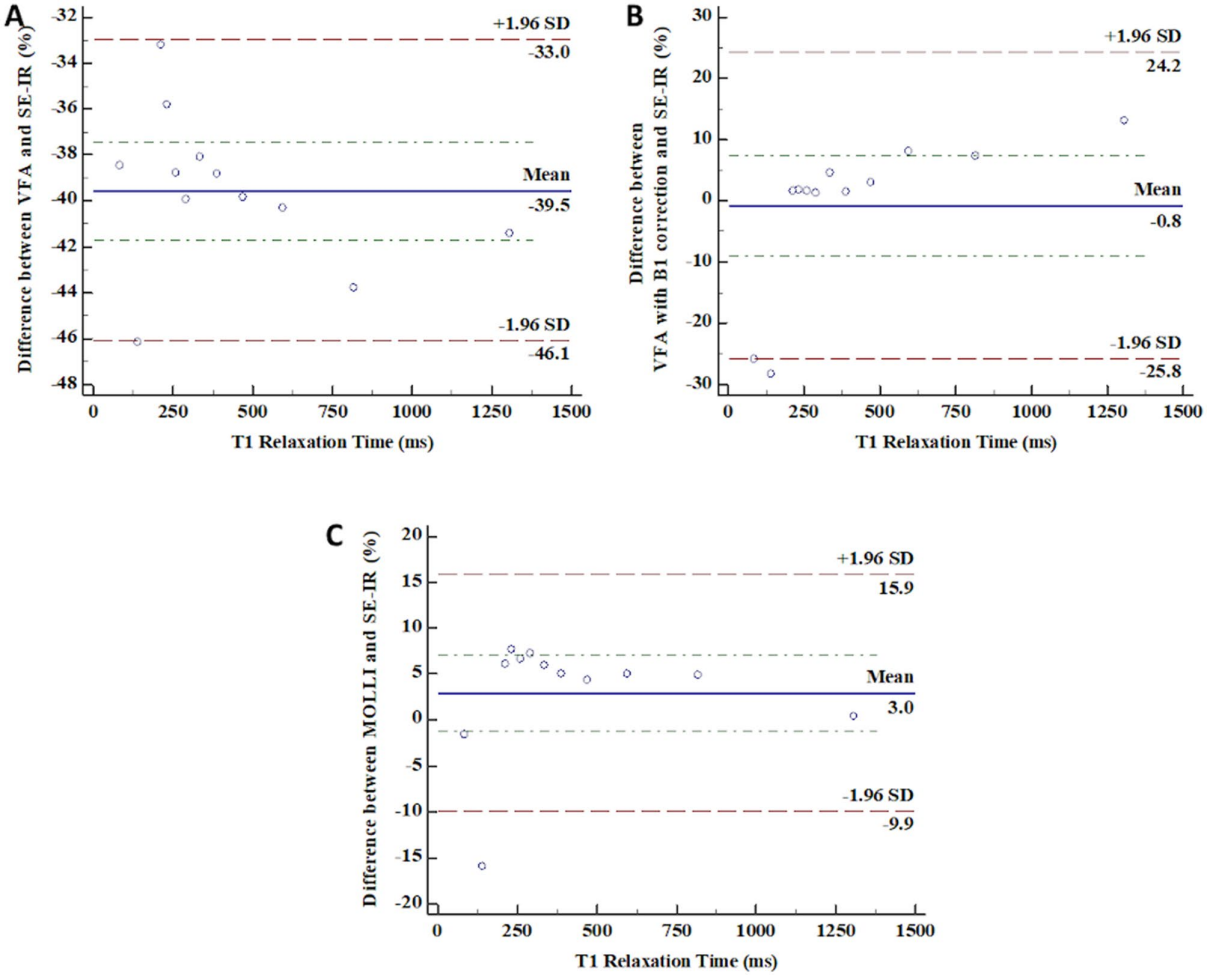
Bland–Altman Curve between VFA and SE-IR, VFA with B1 correction and SE-IR, and MOLLI and SE-IR. (**A**) Bland–Altman Curve between VFA and SE-IR (mean difference = − 39.5%, 95% LOA = − 46.1–− 33.0%). (**B**) Bland–Altman Curve between VFA with B1 correction and SE-IR (mean difference = − 0.8%, 95% LOA = − 25.8–24.2%). (**C**) Bland–Altman Curve between MOLLI and SE-IR (mean difference = 3.0%, 95% LOA = − 1.2–7.2%).

The T1 relaxation times measured by four different sequences according to the concentration of Gadoterate meglumine (Dotarem; Guerbet, Aulnay-sous-Bois, France) are shown in Table 1. T1 relaxivity, which is defined as the inverse of T1 relaxation time, revealed a linear correlation with the concentration of contrast (Pearson correlation coefficient [PCC] = 0.993, 95% confidential interval [CI] 0.975–0.998, *p* < 0.001) (Fig. 1). The T1 relaxation time obtained by VFA with B1 correction and MOLLI methods showed an excellent correlation with the SE-IR method (PCC = 0.999, 95% CI 0.996–1.000 for VFA vs. SE-IR; PCC = 0.999, 95% CI 0.998–1.000 for VFA with B1 correction vs. SE-IR; PCC = 0.999, 95% CI 0.997–1.000 for MOLLI vs. SE-IR; *p* < 0.001) (Fig. 2). Bland–Altman plot was constructed to evaluate the reliability of MOLLI and VFA sequences. The mean difference between VFA and SE-IR sequences was − 39.5% (95% CI − 41.7–− 37.4%; lower and upper limit of agreement (LOA) = − 46.1–33.0%). The mean difference between SE-IR and VFA with B1 correction sequence was − 0.8% (95% CI − 8.9–7.3%; lower and upper LOA − 25.8–24.2%). The mean difference between SE-IR and MOLLI sequence was 3.0% (95% CI − 1.2–7.2%; lower and upper LO − 9.9–15.9%) (Fig. 3). The ICC between VFA and SE-IR was 0.845 (95% CI − 0.017–0.964). The ICC between T1 relaxation times obtained by VFA with B1 correction and SE-IR was 0.994 ms (95% CI 0.980–0.998%). The ICC between T1 relaxation times obtained by MOLLI and SE-IR was 0.999 (95% CI 0.991–1.000%).

**Table 1:** T1 relaxation times measured by four different sequences in the phantom study.

Concentration of Gadoterate meglumine (mmol/L)	T1 Relaxation time by SE-IR (ms)	T1 Relaxation time by VFA (ms)	T1 Relaxation time by VFA with B1 correction (ms)	T1 Relaxation time by MOLLI (ms)
0.1	1305	765	1477	1310
0.2	817	460	878	858
0.3	595	355	643	625
0.4	470	283	485	491
0.5	388	237	394	408
0.6	335	208	351	355
0.7	290	175	294	312
0.8	261	160	265	278
0.9	233	150	237	251
1.0	214	143	217	227
2.0	142	77	102	120
3.0	84	52	63	83

*SE-IR* spin-echo inversion recovery, *VFA* variable flip angle, *MOLLI* modified look-locker inversion recovery.

The range of CV was 0.5 – 1.5% in three consecutive measurements of T1 relaxation times by the MOLLI sequence. The ICC for the three measurements of the MOLLI sequence was 1.000 (95% CI 1.000–1.000%).

### Clinical data

Total 20 children and young adults (M: F = 12: 8; mean age = 20.9 ± 6.6 years; range, 8–29 years) were included in our study. Among them, ten patients had a history of total correction for Tetralogy of Fallot, three patients had a history of valvuloplasty, three patients had arterial switching surgery for correction of transposition of the great arteries, and the remaining four patients had a history of cardiac transplant, Fontan operation, ventricular septal defect repair, and surgery for total anomalous pulmonary venous return.

**Figure 4:**
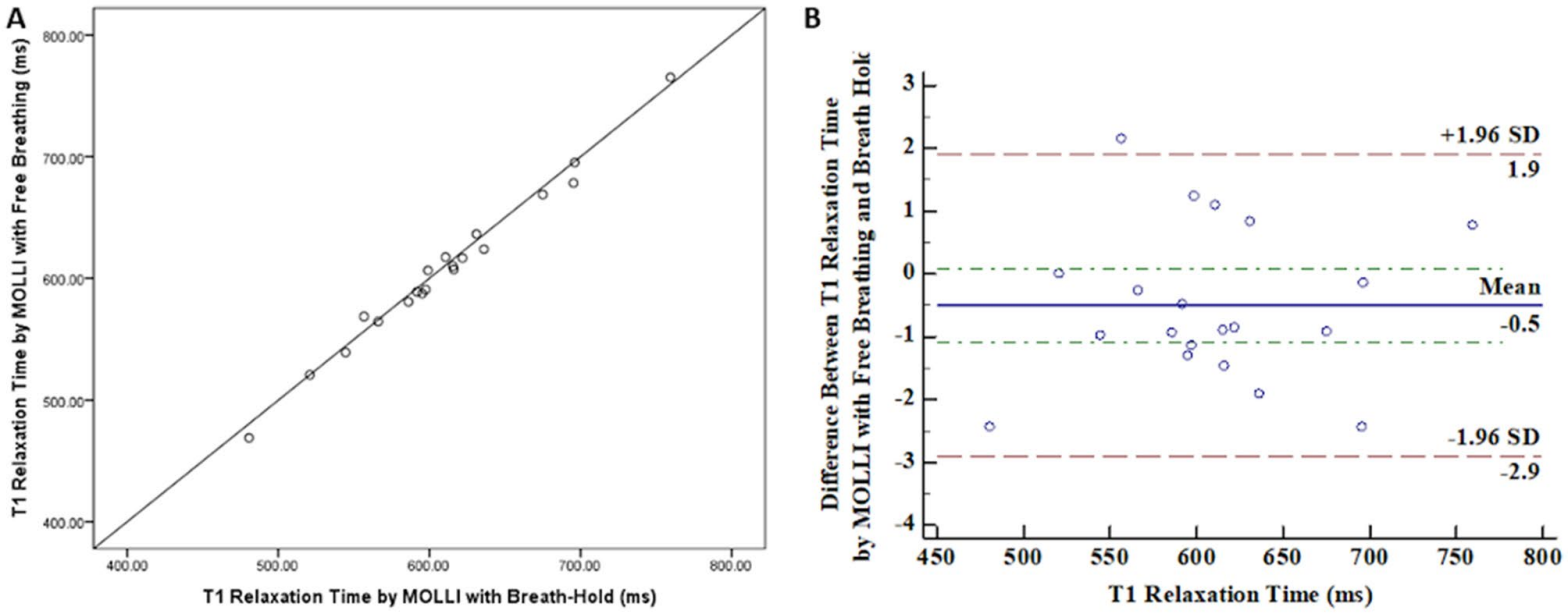
Comparison between free breathing and breath-hold methods in MOLLI sequences. (**A**) The correlation between T1 relaxation time obtained by MOLLI sequence with free breathing and breath-hold methods (Pearson correlation coefficient = 0.993, p < 0.001). (**B**) Bland–Altman Curve between free breathing and breath-hold methods in MOLLI sequences (mean difference = − 0.5%, 95% LOA = − 2.9–1.9%).

The mean values of hepatic T1 relaxation time obtained from the free breathing and breath-hold techniques were 606.7 ± 64.5 ms (range 468.9–765.2 ms) and 609.8 ± 64.0 ms (range 480.6–759.5), respectively. In the inter-observer study, the measurement of T1 relaxation time with free breathing and breath-hold technique revealed an excellent agreement (ICC = 0.999 for both methods). The mean value of the hepatic T1 relaxation time was higher in the breath-holding technique, but it was not statistically significant (*p* = 0.083). The T1 relaxation time obtained by the free breathing and breath-holding technique revealed an excellent correlation (Pearson correlation coefficient; 0.993, *p* < 0.001) (Fig. 4). The percentage of absolute difference ((T1 relation time from free breathing – T1 relaxation time from breath hold)/T1 relaxation time from breath hold × 100), was 1.1% (range 0.01 to 2.4%). The Bland–Altman plot between the free breathing and breath-hold technique revealed that the mean difference in T1 relaxation time was − 3.0 ms (− 0.5%). The range of 95% LOA was − 17.6 to 11.5 ms (− 2.9 to 1.9%). The ICC was 0.996, and the 95% confidential interval of ICC was 0.990 to 0.999.

## Discussion

Our study is the first one to our knowledge in liver imaging compares the breath-hold and free breathing method of hepatic T1 mapping using MOLLI sequence. MOLLI sequence is a robust technique that has already been applied in the clinical settings, especially for cardiac imaging. Although there has been an attempt to apply free-breathing MOLLI sequence in cardiac imaging, any study about hepatic T1 mapping using free-breathing MOLLI sequence has not yet been reported as far as we know^22^.

In our phantom study, MOLLI sequence showed excellent accuracy and reproducibility for T1 mapping. In previous studies investigating normative T1 relaxation times of abdominal and pelvis tissues, those of healthy adults and children were distributed within 300–1500 ms^17,19^. Among them, the T1 relaxation time of the liver, the subject of this study were 586 ± 39 ms, 569 ± 39 ms in adults and children, respectively^17,19^.Therefore, to evaluate the accuracy of the T1 relaxation times measured by each sequence, a phantom with wide range of T1 relaxation time including 500–600 ms were used in our study. In terms of accuracy, the VFA, VFA with B1 correction and MOLLI sequences showed good correlation with the reference standard of the T1 relaxation times measured by SE-IR^19^. However, the T1 relaxation times obtained by the VFA method revealed an under-estimation of T1 values compared to the SE-IR method in our study. VFA without B1 correction is more sensitive to B1 heterogeneity and prone to errors^23^. In our study, both VFA with B1 correction and MOLLI sequences were comparable to the standard SE-IR method. The mean difference from the reference standard was smaller when VFA with B1 correction was used than when using MOLLI sequence (− 0.8% vs. 3.0%). However, Bland–Altman plot showed a proportional error in VFA with B1 correction (Fig. 3). Also, in the 300–1500 ms range where the T1 values of the intra-abdominal organs and tissues were distributed, the difference was smaller in MOLLI sequence than in VFA with B1 correction (4.2% vs. 6.1%)^17,19^. In addition, the T1 relaxation time obtained by MOLLI sequence revealed excellent reproducibility in repetitive examination and measurement. These findings suggest that MOLLI sequence is comparable to the standard SE-IR method and it can be applied to obtain T1 relaxation times in abdominal organs. Furthermore, the MOLLI sequence had a shorter acquisition time than the VFA with B1 correction method. In pediatric patients, sedation is often necessary and only a short scan time is allowed for MRI. The given results of phantom study demonstrate that the MOLLI sequence could be more adequate to obtain T1 relaxation time instead of reference standard method, SE-IR, than any other methods.

The scan time of the MOLLI sequence used in our study was 12 s. It is short enough to acquire T1 maps during single breath-hold, but still problematic for pediatric patients. The classic MOLLI sequence established in myocardial MRI requires breath-holding to avoid the misregistration caused by respiratory motion^22^. The breath-hold technique is also preferred to obtain an accurate hepatic T1 map; however, children, as well as some adult patients, are unable to voluntarily hold their breaths in actual practice. Generally, infants and most children underwent MR imaging in deep sedation or under anesthesia.

Cassinotto et al. demonstrated that T1 relaxation valuesusing 1.5 T MR unit in adult cirrhotic liver group with Child–Pugh class A and normal control groups were 500 ± 79 and 574 ± 84 ms, respectively^5^. In another previous study, Heye et al. showed that T1 relaxation valueusing 1.5 T MR machine in adult cirrhotic liver group and normal control group were 678 ± 45 and 852 ± 132 ms, respectively^6^. In a study with healthy children, hepatic T1 relaxation values with using 1.5 T MR machine was 581 ± 64 ms^17^. In our study, the hepatic T1 values obtained with MOLLI sequence by free breathing (606.7 ± 64.5 ms) and breath-holding (609.8 ± 64.0 ms) techniques showed no significant differences. The absolute difference between these two methods was 0.055 to 17.0 ms (mean = 6.7 ms). Although we did not investigate the difference of hepatic T1 valuesusing MOLLI sequence between cirrhotic liver group and normal control group in our study, we think the difference of T1 values between free breathing and breath-hold techniques is small enough to be accepted in evaluating liver fibrosis, referring to the previous data which measured the hepatic T1 values of cirrhotic and normal liver^5,6^. Therefore, we believe MOLLI with free-breathing technique could be acceptable for the evaluation of hepatic fibrosis in children and young adults.

There are several reasons why the MOLLI sequence has strengths in free breathing. The bSSFP sequence we used in the MOLLI technique has a short TR and read-out duration with a high signal to noise ratio; thus it can be used to rapidly acquire images with fewer motion^13^. In addition, the use of a non-selective IR pulse in the MOLLI sequence may help to reduce motion artifacts. Moreover, we routinely applya fully automated non-rigid motion correction. In a previous study, the most important factor to apply free-breathing MOLLI is image registration^22^. The inline motion correction can reduce the misregistration of each pixel in the acquired images during the three-consecutive look-locker experiments. For image registration, more images were obtained when free-breathing MOLLI was applied than when breath-hold MOLLI was used in a previous study^22^. In our study, it was possible to generate a comparable T1 map using free-breathing MOLLI with the same number of images as breath-hold sequence. Finally, hepatic fibrosis is a diffuse disease that involves the entire liver parenchyma; thus, it is expected that any misregistration caused by the free breathing method might not have a significant influence on the hepatic T1 values of the patient.

There were several limitations in our study. First, we did not evaluate the degree of hepatic fibrosis of the patients by pathology or laboratory results. Thus, there are no results about the changes of T1 values according to degree of hepatic fibrosis in our study. In future work, it is necessary to evaluate the differences in hepatic T1 values obtained by free breathing and breath-hold methods in patients who have different degrees of hepatic fibrosis. Second, the patients included in our study are relatively older pediatric patients. It is difficult to say that our results represent the difference of hepatic T1 values according to the two different methods in patients of very young age. However, in very young children it is difficult to obtain hepatic T1 value with breath-hold in practice. Third, the small study population is another limitation of our study.

In conclusion, MOLLI sequence can be applied to obtain hepatic T1 relaxation times comparable to the values obtained using the standard SE-IR method. The hepatic T1 relaxation times measured using the MOLLI technique with free breathing were comparable to those obtained with breath-holding in children and young adults.

## Methods

This retrospective study was approved by the institutional review board of Seoul National University Hospital (IRB no. 1902-053-1008), and the requirement for informed consent was waived. All methods were performed in accordance with the relevant guidelines and regulations.

### Phantom preparation

**Figure 5:**
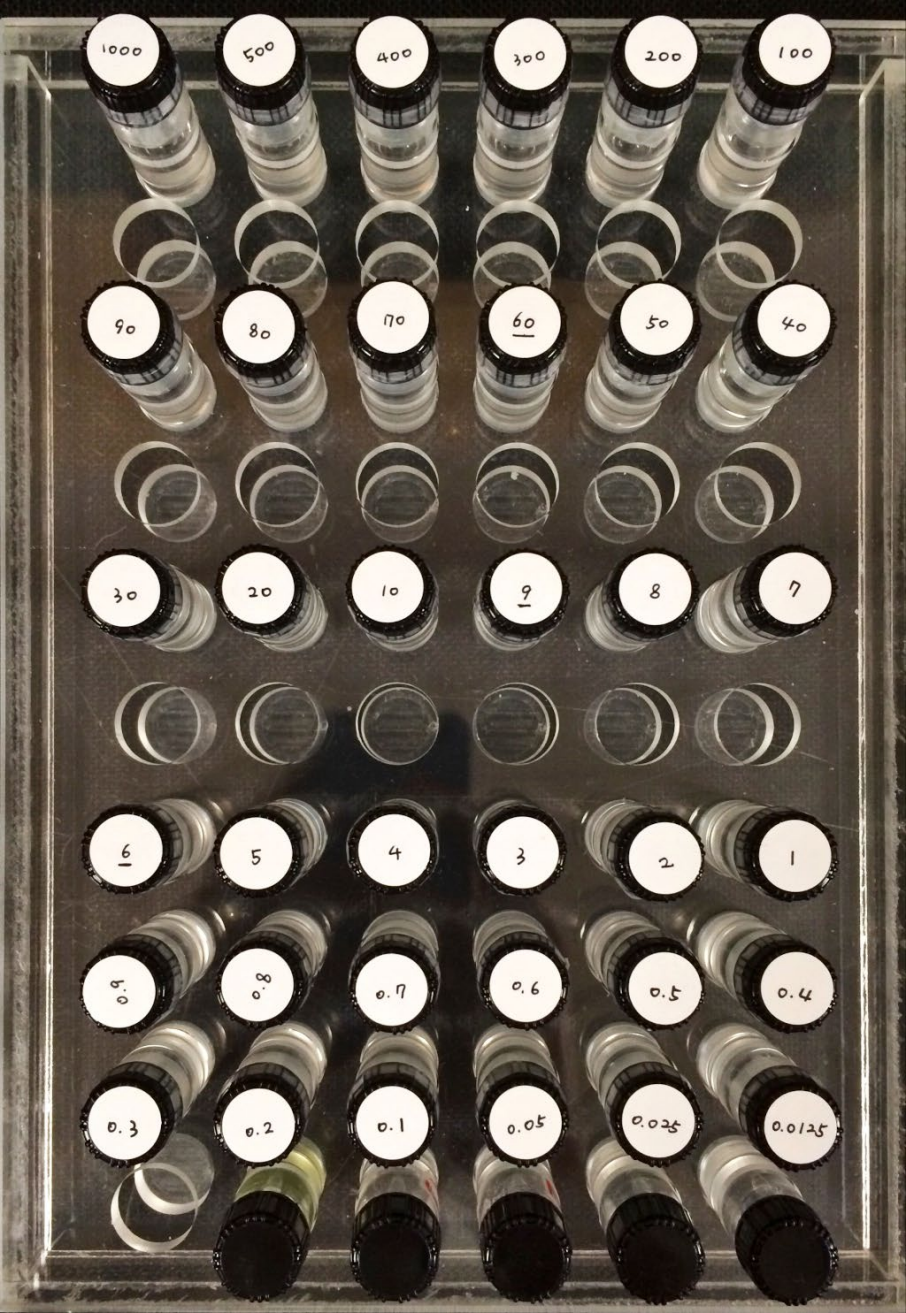
Phantom objects. Phantom tubes containing multiple vials of various Gadoterate meglumine concentrations.

To evaluate the accuracy of T1 values obtained with the MOLLI sequence, we prepared an acrylic box of twelve cylindrical tubes (3 × 3 × 6 cm) with different concentrations of gadoterate meglumine (0.1, 0.2, 0.3, 0.4, 0.5, 0.6, 0.7, 0.8, 0.9, 1, 2, and 3 mmol/L, respectively) (Fig. 5). We scanned the phantom with four different methods of T1 mapping, which included 2D SE-IR, VFA, VFA with B1 correction, and MOLLI sequences. To evaluate the reproducibility of T1 values obtained by the MOLLI sequence, we scanned these phantom tubes three times with the MOLLI sequence.

A series of IR sequences was obtained using the following parameters: repetition time (TR) = 2550 ms; echo time (TE) = 10 ms; Flip angle (FA) = 90°; field of view (FOV) = 260 × 260 mm^2^; matrix = 256 × 256 pixels; slice thickness = 8.0 mm; inversion time (TI) = 50, 400, 1100, and 2500 ms; total acquisition time = 43 min and 48 s.

For the VFA method, we used three different flip angles to obtain the T1 map with VFA sequence. Three dimensional (3D) volumetric interpolated breath-hold examination (VIBE) was used and the parameters used were as follows: TR = 5.2 ms; TE = 1.76 ms; flip angle (FA) = 2°, 5°, 15°; FOV = 195 × 260 mm^2^; matrix = 160 × 320 pixels; slice thickness = 4.0 mm; generalized auto-calibrating partial parallel acquisition (GRAPPA) acceleration factor = 2; total acquisition time = 15 s.

For the VFA with B1 correction method, the dual flip angle 3D-VIBE sequence was used. The B1 map was obtained before the acquisition of T1 mapping to correct B1 inhomogeneity. The following parameters were used: TR = 15 ms; TE = 1.7 ms; flip angle (FA) = 5°, 26°; FOV = 260 × 260 mm^2^; matrix = 384 × 384 pixels; slice thickness = 3.0 mm; GRAPPA acceleration factor = 2; acquisition time = 67 s. B1 map sequence based on the spin-echo type, with a slice selective excitation and two refocusing pulses, which generates a spin-echo and a stimulated-echo was used to correct B1 inhomogeneity. The detailed imaging parameters of the B1 map sequence and calculation of T1 values with corrected B1 are provided in Supplementary Information.

**Figure 6:**
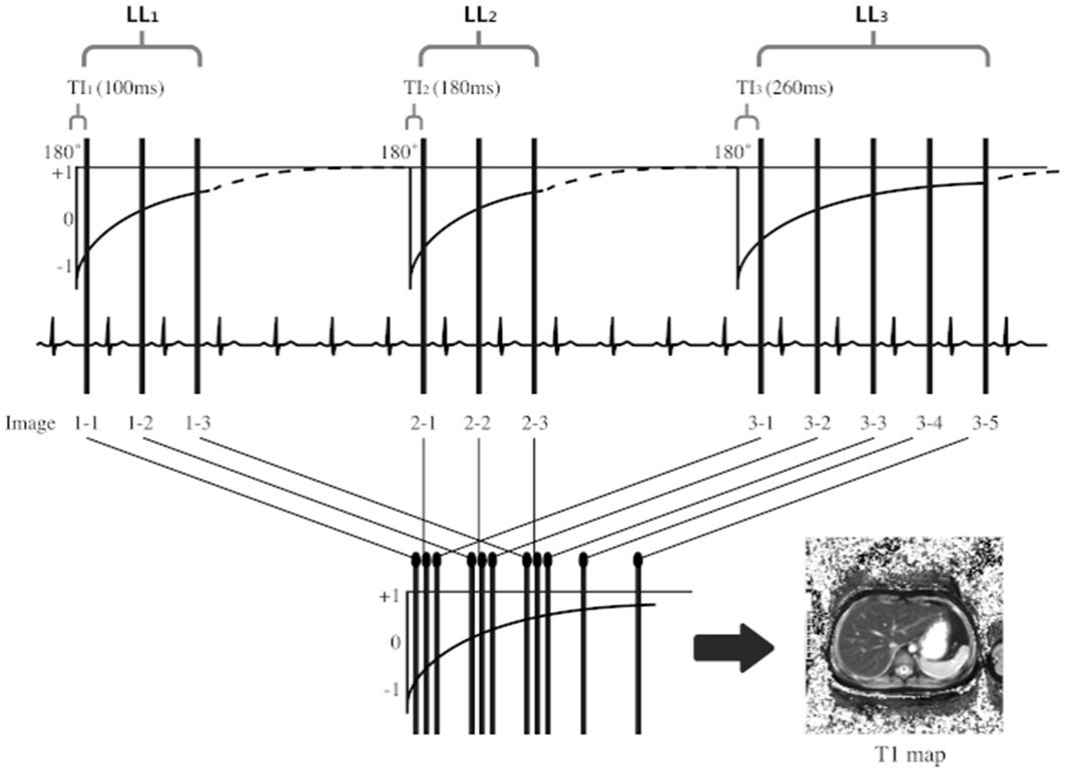
Modified Look-Locker Inversion Recovery (MOLLI) pulse sequence with 3(4)3(4)5(0) scheme. Three sets of look-locker experiments were performed with increasing inversion times. Three, three, and five images were acquired after the first, second, and third inversion pulses, respectively with four RR intervals between each inversion pulse for T1 recovery.

The MOLLI sequence consisted of three inversion recovery-prepared look-locker pulses, and the imaging parameters used were as follows: 2D- single-shot balanced steady-state free-precession (bSSFP); TR/TE = 2.32/1.03 ms; flip angle = 35°; number of excitations = 1.0; field of view (FOV) = 349 × 349 mm^2^; matrix = 192 × 134 pixels; minimum inversion time (TI) = 100 ms; TI increment between inversions = 80 ms; slice thickness = 8 mm; number of inversions = 3; number of acquisitions after an inversion pulse = 3, 3, 5; number of recoveries after each inversion pulse = 4, 4, 0; acquisition time = 12 s. A fully automated non-rigid motion correction was applied to register the individual images (Fig. 6). The cardiac cycle synchronization was performed with pulse oximetry, obtained through a sensor placed on patient`s index finger.

After this, inline fitting was performed using a mono-exponential, three-parameter fit, and T1 maps were automatically calculated using work-in-progress software provided by the vendor (Siemens Healthcare, Erlangen, Germany).

All MR images were obtained at a 1.5 unit (Avanto, Siemens Healthcare, Erlangen, Germany) using a 12-channel head coil and 6-channel phased array body coil. The gadoterate meglumine phantom was scanned under a constant room temperature of 20–21 ℃.

### T1 mapping with MOLLI sequence in patients

#### Study population

We obtained liver MRI including MOLLI sequences in combination with cardiac MRI in patient who had a history of congenital heart disease. Additional liver MRI was performed during cardiac MRI in patients who had liver function abnormality with suspicion of congestive hepatopathy. We obtained MOLLI sequences twice (free breathing and breath-hold techniques) in 22 patients who could hold their breath for 20 s. We excluded 2 patients who had focal hepatic masess in liver MRI.

#### MR imaging acquisition for patients

The MOLLI sequence were obtained without contrast enhancement. One axial slice was chosen at the levels showing the largest area of the liver parenchyma among the baseline, axial, T1-weighted images. The imaging parameters were the same as those used for the phantom study. T1 maps were acquired twice in each patient, one with free breathing and the other with breath-hold technique.

### Data analysis

The measurements of T1 relaxation times were achieved by the manual drawing of the regions of interest (ROIs) on the automatically generated T1 maps. In the phantom study, a radiologist (Y. J. C.) with eight years of clinical experience measured T1 values three times in each cylinder on axial T1 maps by drawing approximately 100 mm^2^-sized circular ROIs in the center of each cylinder. The mean T1 values of each sequence were used for the analysis. The reference standard determination was based on axial, single-section imaging of the phantom midpoint with SE-IR sequence.

**Figure 7:**
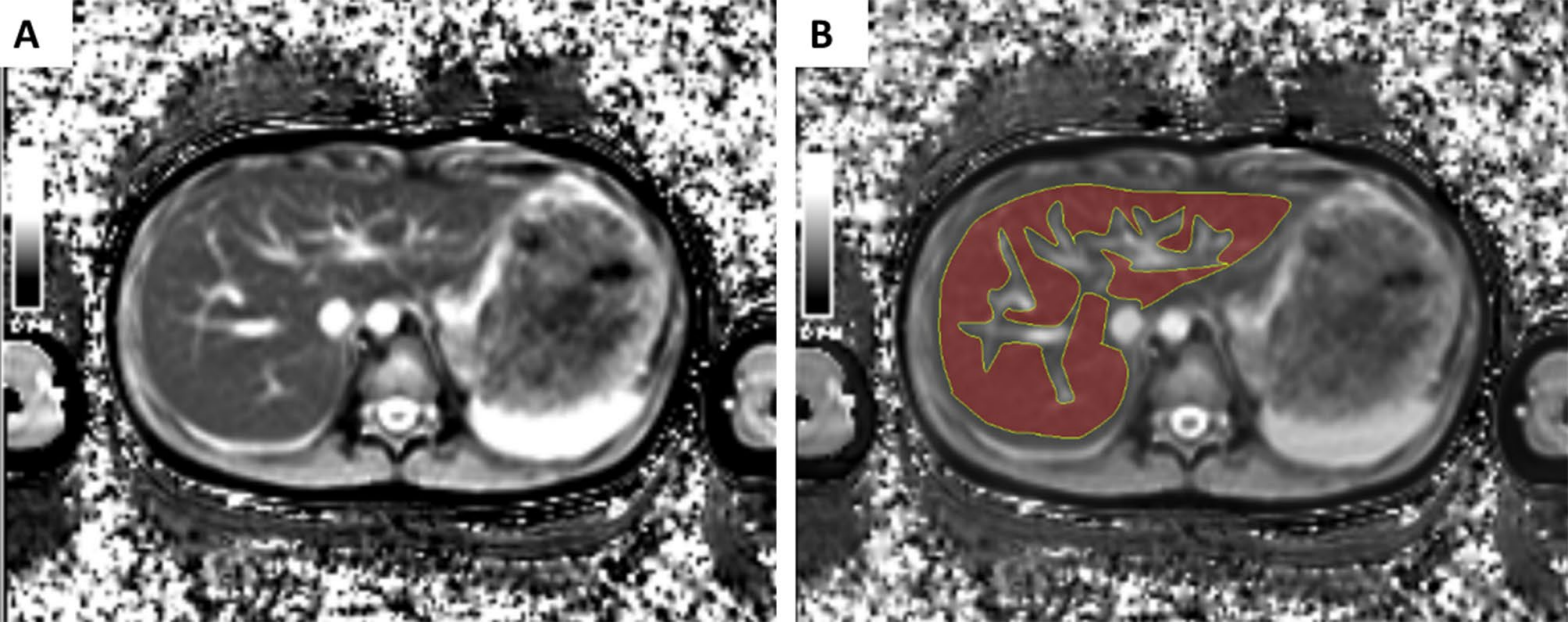
Example of a hepatic T1 map obtained by MOLLI sequence and the measurement of T1 relaxation time. (**A**) T1 map obtained by MOLLI sequence in a 13-year-old boy. (**B**) An example of the measurement of T1 relaxation time by the manual drawing of ROI with the exclusion of hepatic vasculature.

In the human study, the measurement of the hepatic T1 relaxation time was performed by two independent radiologists, (Y. J. C. and S. H. L, 8 years of experience) who performed manual drawings of freehand ROIs on the T1 maps with the exclusion of identifiable vascular structures, including hepatic and portal veins (Fig. 7). As obtained in the phantom study, the mean T1 relaxation times were also calculated.

### Statistical analysis

To evaluate the correlation between reference T1 relaxation times obtained by the SE-IR method and the concentration of Gadoterate meglumine, we employed the Pearson correlation analysis; which was also used to identify the correlation between the standard SE-IR methods and the three different methods. We calculated the intraclass correlation coefficient (ICC) and constructed a Bland–Altman plot to evaluate the absolute agreement among the T1 relaxation times of the four different techniques used in the phantom study. The reliability and reproducibility of the T1 MOLLI sequence were evaluated by using the within-subject coefficient of variance (CV) and ICC.

The mean hepatic T1 relaxation times were compared between the free breathing and breath-hold techniques by using paired t-test and Pearson correlation analysis. For evaluating the absolute agreement of T1 values between free breathing and breath-hold MOLLI techniques, we calculated the two-way ICC based on a single-rating, absolute-agreement, and 2-way random effects model. A Bland–Altman plot was constructed, and a 95% limit of agreement was calculated.

A P-value of less than 0.05 was determined as statistically significant. All statistical analyses were performed using commercially available software (MedCalc, Version 12, MedCalc Software, Mariakerke, Belgium; IBM SPSS Statistics, Version 23.0, SPSS Inc., Armonk, NY, USA).

### IRB statement

This retrospective study was approved by the institutional review board, which waived the need for patient informed consent.

## Supplementary information

Supplementary Information.
