# Supplementary material for: Validation and feasibility of liver T1 mapping using free breathing MOLLI sequence in children and young adults
Source: Sci Rep. 2020 Oct 27;10:18390. doi: 10.1038/s41598-020-74717-2 (PMC7591907; doi:10.1038/s41598-020-74717-2)
Supplement: Supplementary file 1 — Supplementary Information. [file 41598_2020_74717_MOESM1_ESM.pdf]

Supplementary Information

Title: Validation and Feasibility of Liver T1 Mapping Using Free Breathing MOLLI Sequence in Children and Young Adults.

Authors: Yeon Jin Cho, M.D., Woo Sun Kim, M.D., Ph.D.,Young Hun Choi, M.D., Seul Bee Lee, M.D., SeungHyun Lee, M.D., Jung-Eun Cheon, M.D., Ph.D., MunYoung Paek, SeungTae Woo, Ph.D.

S1. Imaging Parameters of B1 maps

Two images were obtained by the B1 map sequence. The first one contained the magnitude information (sum of squares of spin-echo and stimulated echo data), and the second one contained the flip angle information, encoded as a phase image where the range from 0° to 180° was mapped to pixel values from 0 to 1800. Dividing the pixel value by 10 provides the local flip angle in degree.

The parameters for the B1 map were as follows: repetition time (TR) = 1000 ms; echo time (TE) = 14 ms; flip angles= 90°; and section thickness =5 mm with an intersection gap of 5 mm; field of view = 250 mm × 250 mm; matrix = 32 × 32; acquisition time = 37 seconds.

S2. Calculation of T1 values and T1 values with corrected B1

T1 values is calculated on a per pixel basis using the following formula:

$$\mathrm{T1(x,y)} = \frac{TR}{\ln\left[\dfrac{\sin(\alpha 1)\cos(\alpha 2) - \dfrac{S1(x,y)}{S2(x,y)}\sin(\alpha 2)\cos(\alpha 1)}{\sin(\alpha 1)\dfrac{S1(x,y)}{S2(x,y)}\sin(\alpha 2)}\right]}$$

S1(x, y), S2(x, y) pixel values correspond to different flip angles. T1 value with the corrected B1 was calculated using the following formula:

$$\mathrm{B1\ corrected\ T1(x,y)} = \frac{TR}{\ln\left[\dfrac{\sin(\alpha 1\cdot c(x,y))\cos(\alpha 2\cdot c(x,y)) - \dfrac{S1(x,y)}{S2(x,y)}\sin(\alpha 2\cdot c(x,y))\cos(\alpha 1\cdot c(x,y))}{\sin(\alpha 1\cdot c(x,y))\dfrac{S1(x,y)}{S2(x,y)}\sin(\alpha 2\cdot c(x,y))}\right]}$$

where c(x, y) refers to the relative B1 inhomogeneity for the pixel position calculated from the B1

map.
